# Supplementary material for: Identification of photoperiod-induced specific miRNAs in the adrenal glands of Sunite sheep (Ovis aries)
Source: Front Vet Sci. 2022 Jul 22;9:888207. doi: 10.3389/fvets.2022.888207 (PMC9354845; doi:10.3389/fvets.2022.888207)
Supplement: Supplementary file 1 [file Data_Sheet_1.ZIP › Supplementary file/miRDeep2 file.zip!/miRDeep2 file/Know_Structure/SP42A3/oar-mir-29b-2.pdf]

miRBase precursor : oar-mir-29b-2
Total read count : 10597
oar-miR-29b read count : 9670
remaining reads : 927

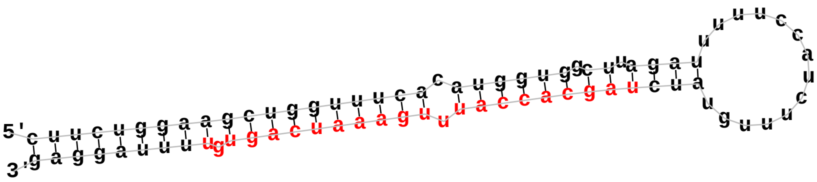

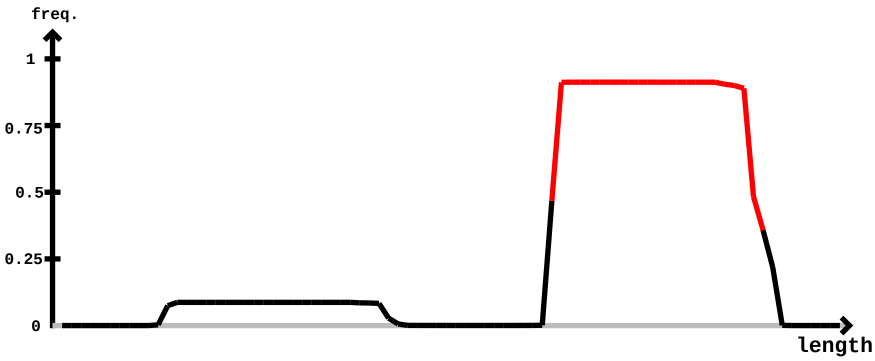

oar-miR-29b

```
5'- cuucuggaagcugguuucacaugguggcuuagauuuuuccaucuuuguaucuagcaccauuugaaaucaguguuuuaggag   -3'    exp
    (((((((((((((((((((.((((((.((.((((..............)))))))))))).)))))))))).)))))))))    reads   mm   sample
    .........gcugguuucacaugguggcuu...................................................    7       0    seq
    .........gcugguuucacaugguggcuuag.................................................    1       0    seq
    .........gcugguuucacaugguggcuuaga................................................    22      0    seq
    .........gcugguuucacaugguggcuuagauuuuuc..........................................    1       0    seq
    ..........cugguuucacaugguggcuu...................................................    11      0    seq
    ..........cugguuucacaugguggcuua..................................................    7       0    seq
    ..........cugguuucacaugguggcuuag.................................................    7       0    seq
    ..........cugguuucacaugguggcuuaga................................................    513     0    seq
    ..........cugguuucacaugguggcuuagau...............................................    181     0    seq
    ..........cugguuucacaugguggcuuagauu..............................................    40      0    seq
    ..........cugguuucacaugguggcuuagauuu.............................................    2       0    seq
    ...........ugguuucacaugguggcuu...................................................    1       0    seq
    ...........ugguuucacaugguggcuuag.................................................    7       0    seq
    ...........ugguuucacaugguggcuuaga................................................    55      0    seq
    ...........ugguuucacaugguggcuuagau...............................................    51      0    seq
    ...........ugguuucacaugguggcuuagauu..............................................    16      0    seq
    .................................uuuuuccaucuuuguauc..............................    2       0    seq
    .................................uuuuuccaucuuuguaucG.............................    1       1    seq
    ..................................uuuuccaucuuuguaucuagcaccauu....................    1       0    seq
    ...............................................uaucuagcaccauuugaaauca............    1       0    seq
    .................................................ucuagcaccauuugaaaucagu..........    9       0    seq
    ..................................................cuagcaccauuugaaau..............    1       0    seq
    ..................................................cuagcaccauuugaaauc.............    34      0    seq
    ..................................................cuaCcaccauuugaaauc.............    1       1    seq
    ..................................................cuagcaccauuugaaauca............    13      0    seq
    ..................................................cuagcaccauuugaaaucag...........    20      0    seq
    ..................................................cuagcaccauuugaaaucGg...........    2       1    seq
    ..................................................cuagcaccauuugaaaucGgu..........    21      1    seq
    ..................................................cuagcaccauuugaaaucagu..........    2595    0    seq
    ..................................................cuagcaccauuugaaaucagug.........    1101    0    seq
    ..................................................cuagcaccauuugaaaucagugu........    1145    0    seq
    ..................................................cuagcaccauuugaaaucaguguu.......    6       0    seq
    ...................................................uagcaccauuugaaauU.............    3       1    seq
    ...................................................uagcaccauuugaaauc.............    42      0    seq
    ...................................................uagcaccauuugaaauA.............    1       1    seq
```

oar-miR-29b

cuucuggaagcugguuucacaugguggcuuagauuuuuccaucuuuguaucuagcaccauuugaaaucaguguuuuaggag			
....uagcaccauuugaaaucG............	22	1	seq
....uagcaccauuugaaauca............	14	0	seq
....uagcaccauuugaaaucag...........	17	0	seq
....uagcaccauuugaaaucGg...........	71	1	seq
....uagcaccauuugaaaucGgu..........	515	1	seq
....uagcaccauuugaaaucagu..........	1161	0	seq
....uagcaccauuugaaaucagug.........	238	0	seq
....uagcaccauuugaaaucagugu........	318	0	seq
....uagcaccauuugaaaucaguguu.......	2304	0	seq
....uagcaccauuugaaaucaguguuu......	9	0	seq
....uagcaccauuugaaaucaguguuuu.....	1	0	seq
....agcaccauuugaaaucagu..........	3	0	seq
....agcaccauuugaaaucaguguu.......	2	0	seq
....gcaccauuugaaaucaguguu.......	1	0	seq
